# A Trans Youth of Color Study to Measure Health and Wellness: Protocol for a Longitudinal Observation Study

**DOI:** 10.2196/39207

**Published:** 2022-11-07

**Authors:** Sam Calvetti, Joshua A Rusow, Jacqueline Lewis, Amarah Martinez, Lindsay Slay, Bethany C Bray, Jeremy T Goldbach, Michele D Kipke

**Affiliations:** 1 Children's Hospital Los Angeles Los Angeles, CA United States; 2 The Brown School Washington University in St Louis St Louis, MO United States; 3 Health Services Los Angeles LGBT Center Los Angeles, CA United States; 4 HIV & Biomedical Department Wesley Health Centers JWCH Institute Palmdale, CA United States; 5 Institute for Health Research and Policy University of Illinois Chicago Chicago, IL United States; 6 Department of Pediatrics Keck School of Medicine University of Southern California Los Angeles, CA United States

**Keywords:** AIDS virus, HIV, cohort study, gender minority, transgender youth

## Abstract

**Background:**

Growing research on transgender youth is accounting for the variety of ways in which young people define their genders and sexualities. Because of this growing representation, more research is needed to understand how intersectional identities and stigma affect risk for HIV acquisition along the HIV care continuum and engagement in mental and physical health care. Little is known about accessibility to HIV-related prevention services of nonbinary and transmasculine youth, and further understanding of the impacts on transfeminine people—those who have historically faced the highest prevalence of HIV positivity—is crucial.

**Objective:**

The overarching aims of the Trans Youth of Color Study are to conduct longitudinal research with a cohort of transgender minority youth (TGMY), explore factors that aid in the prevention of new HIV infection and transmission, and reduce HIV- and AIDS-related disparities by focusing on successful engagement in care. Findings from this research will be used to inform the development of new interventions designed to engage TGMY in the HIV prevention and care continua.

**Methods:**

Longitudinal research (baseline and follow-up assessments every 6 months for 3 waves of data collection) followed a cohort (N=108) of transgender youth of color recruited in Los Angeles, California, United States. Participants were recruited using multiple community-informed strategies, such as from local venues, social media, and participant referral. In addition to self-report surveys, urine was collected to assess recent use of illicit drugs, and blood, rectal, and throat swabs were collected to test for current sexually transmitted infection and HIV infection. Additional blood and plasma samples (10 mL for 4 aliquots and 1 pellet) were collected and stored for future research.

**Results:**

Participants in the Trans Youth of Color Study were recruited between May 25, 2018, and December 7, 2018. Baseline and longitudinal data are being analyzed as of August 2022.

**Conclusions:**

The findings from this research will inform adaptations to existing evidence-based HIV prevention interventions and help to guide new interventions designed to engage TGMY, especially those who are Black, Indigenous, or people of color, in the HIV prevention and care continua.

**International Registered Report Identifier (IRRID):**

DERR1-10.2196/39207

## Introduction

### Intersecting Identities Among Transgender Youth

The lesbian, gay, bisexual, transgender, queer, intersex, and asexual (LGBTQIA) community comprises an incredibly diverse community of people from many races, ethnicities, religions, and socioeconomic backgrounds who also share a wide variety of sexual identity– and gender-related experiences. Specifically, transgender individuals seem to experience unique and complex patterns of gender-related experiences throughout the life course [1,2].

Some transgender people may identify with a gender that differs from the sex that they were assigned at birth, and others may identify as being beyond the gender binary, such as nonbinary, genderqueer, gender nonconforming, agender, or other combinations of identities that reflect their personal experience [3].

Data on the prevalence of nonbinary individuals in the transgender community varies across studies, from 52% of 14,320 transgender survey respondents in the United Kingdom to 35% of 27,715 transgender survey respondents in the United States [4,5]. Furthermore, existing data highlight generational differences in nonbinary groups, which illustrate a greater gender-fluid expression among younger individuals [5,6]. However, existing information on these gender minority groups is limited and mostly based on the experiences of White, middle-class populations [7].

### Stigma and Health Disparities Among Transgender Youth

Racial and ethnic minorities who also identify as transgender and nonbinary remain particularly underexplored, and this is evident in the lack of available resources designed to help these individuals achieve a higher quality of life [8,9]. Specifically, among these groups, transgender minority youth (TGMY; ie, transgender and nonbinary people of color aged between 16 and 24 years) seem to represent the greatest opportunity to intervene and improve long-term health behaviors; this is due to a combination of environmental factors that put them at increased risk to be victimized by others, experience internalized transphobia, and have trouble in finding affirming resources; for example, TGMY are 3 times more likely than young men who have sex with men (YMSM) to experience workplace discrimination [10], health care providers report a lack of preparation to care for TGMY, and many institutions lack policies and routine practices to support the needs of transgender patients [11,12].

Because of their experiences with multiple identities—for example, gender identity, sexual identity, and racial and ethnic identity—TGMY face more complex patterns of intersectionality, which may lead to greater experience of various forms of stigma (eg, violence, isolation, and harassment). These experiences, in turn, systemically place stress on TGMY, placing them at higher risk for mental health conditions (eg, depression, anxiety, and suicidality); substance use; and sexual health risk, including HIV transmission and acquisition [2,9,13-17].

Regarding sexual health, data show that 14% of transgender women and 3% of transgender men live with HIV compared with <0.5% of the general American population [18,19]; 36% of transgender women and 23% of transgender men who seroconverted during the period from 2009 to 2014 were aged between 13 and 24 years [14]. Perhaps contributing to these disparities, 25% to 43% of TGMY report experiencing unstable housing or homelessness [20,21], 67% report engaging in sex work [20], and 31% report experiences of sexual violence in the past 12 months [22].

### A Trans Youth of Color Study

Because of this increased risk for violence and victimization and the necessity to enhance our contextual understanding of multiple forms of stigma faced by TGMY in relation to their increased risk for HIV [8], we applied for an administrative supplement to our existing cohort study [23] to longitudinally observe a cohort of TGMY of color from Los Angeles, California, United States. There has been little research highlighting the developmental health trajectories of transgender and nonbinary youth as well as long-term outcomes for overall well-being. This paper describes the process of designing a longitudinal study to address the gaps in our contextual understanding of TGMY of color and their lived experiences.

### Overarching Goal and Specific Aims

The Trans Youth of Color (TRUTH) Study aimed to expand the research of our parent grant, the Healthy Young Men’s (HYM) Cohort Study (U01DA036926). As a supplement to this parent project, the TRUTH Study proposed to recruit a sample of 125 African American and Latinx young transgender women and collect 2 waves of data. Herein, we highlight the process of expanding our eligibility criteria and aims through iterative community-informed research. The overarching aim of the project is to better understand the unique challenges and opportunities regarding engaging these young transgender people in primary care and the HIV prevention care continuum.

The specific aims are as follows:

- Aim 1: Conduct qualitative research (focus groups and one-on-one interviews) with transgender youth of color to better understand what linkage, engagement, retention to primary health, pre-exposure prophylaxis (PrEP), and antiretroviral therapy care and adherence mean to them to identify potential strategies for intervention.
- Aim 2: Characterize transgender youth of color on measures of (1) alcohol and illicit drug use; (2) sexual risk behaviors, including sex work; (3) use of HIV testing and prevention services; (4) incidence of HIV and sexually transmitted infections (STIs); (5) use of hormone therapy (physician prescribed or obtained in other ways); (6) insurance status and access to health care services, including primary care and HIV and AIDS treatment services; (7) engagement in, and use of, health care and HIV and AIDS treatment services; and (8) use of PrEP. We harmonized data collection with the HYM Cohort Study for some measures and administered additional transgender individual–specific measures. This will allow us to compare the responses of transgender youth of color with those of YMSM.
- Aim 3: Identify transgender individual–specific barriers and facilitators to engagement in primary health and HIV-related care, including transgender individuals’ coping and adjustment strategies, gender identity, gender-related stigma, self-esteem, empowerment, lack of culturally competent providers, and use of services for transgender individuals.

### Theoretical Model and Conceptual Framework

Our HYM Cohort Study found that YMSM of color experience the highest rates of risk factors as framed by syndemic theory, which posits that accumulations of health problems can potentially compound and amplify the negative impact of other health problems [24-26]. For YMSM of color, health issues related to alcohol and substance use, intimate partner violence (IPV), depression, and other health care factors affect wellness; in addition, overlapping stigmas such as racism, discrimination, and homophobia are associated with negative health impacts. Within the HYM Cohort Study sample, 87% of the participants reported experiencing racism, 76% reported experiencing homophobia, and 26% tested positive for ≥1 STIs [27]. These experiences have each been found to be significantly associated with misuse of substances and involvement in sexual practices with higher risk for HIV transmission [28,29].

The proposed analyses will examine syndemic risk factors as predictors of HIV infection among transgender youth of color as well as engagement in care, including HIV prevention, testing, and treatment. The TRUTH Study will focus on areas affecting the health and wellness of transgender youth, such as engagement and retention to primary health care, access to PrEP and antiretroviral therapy, alcohol and substance use, and sexual health behaviors. Through understanding the impacts of intersectional stigma on this cohort, we also hope to observe possible facilitators to care and wellness, such as coping and adjustment, self-esteem, community belongingness, and positive transgender identity.

The purpose of this paper is to describe the protocol for the TRUTH Study: the community-informed method of study design, research methods, and longitudinal recruitment and retention.

## Methods

### Consent and Ethics Approval

As a supplemental study to our longitudinal HYM Cohort Study, which involves following a cohort of 450 Black or African American, Latinx, and multiracial YMSM in Los Angeles [23], the TRUTH Study has been reviewed and approved by the institutional review board of Children’s Hospital of Los Angeles (CHLA-14-00279). Herein, we outline the process of identifying eligible TGMY participants, the process of selecting our final sample criteria, obtaining informed consent, and collecting data from the participants.

As a community-oriented research project, an important component of the informed consent process was meeting participants *where they are at*, meaning conducting field-based, face-to-face consent visits at locations most convenient to participants. All participants provided written informed consent after reviewing consent documents with research field staff. Participants were provided with infographics explaining the consent process (assent process for participants aged <18 years) and the process of participating in the project.

All participants were identified, screened for eligibility, and, if eligible, invited to participate in the study, as described in the following sections. All participants provided written informed consent during a face-to-face consenting visit. A certificate of confidentiality was obtained from the National Institute on Drug Abuse, and a waiver of parental consent was obtained for participants aged 16 to 17 years.

### Study Design

Foundational research with the HYM Cohort Study informed the creation and implementation of the TRUTH Study design [23]. Originally, the TRUTH Study proposed to collect two waves of data (baseline and 6-month follow-up assessment). Because of interest in the participant population from our collaborators, scientific committee, and community partners, as well as the demonstrated need for more research on TGMY represented by the TRUTH Study in comparison with other available research, a third wave of data collection was implemented. The cohort consists of 108 TGMY participants. Participants were recruited using multiple community-informed strategies, such as recruitment from public venues, social media, and respondent-driven sampling design described herein.

Modified self-report surveys using scales from the HYM Cohort Study were used to assess social, behavioral, and health concerns specific to TGMY and their intersectional lived experiences. In addition to these measures, data collection also included biological markers for recent illicit substance use via urine analysis, rapid HIV testing, and STI testing. Following our protocol for the original HYM Cohort Study, we collected additional samples of blood (10 mL for 4 aliquots and 1 pellet) and a rectal swab to be stored in a biorepository for future analysis. These samples were collected once during the project [23]. Care was taken to ensure gender-affirming testing environments for the TGMY research participants—specific testing measures used are detailed in the Measures section. The TRUTH Study presents an opportunity for assessing the protective factors that affect social determinants of health, the development and suitability of transgender individual–specific interventions involving evolving biomedical prevention interventions such as PrEP and postexposure prophylaxis, and the impact of affirmation on mental health.

### Study Participants

Although we had initially proposed to recruit young Black or African American or Latinx transgender women, our community advisory board (CAB), youth CAB (YCAB), and scientific advisory group strongly encouraged us to broaden our recruitment strategy to include transgender women, transgender men, and gender nonbinary youth (including gender nonconforming, gender fluid, genderqueer, and gender identities other than cisgender). They also advised us to expand our eligibility to include all Black, Indigenous, and other youth of color. Although much previous research has focused on transgender women and their risk for HIV acquisition because they experience intersectional forms of discrimination, there is growing understanding that research needs to be conducted to understand HIV and STI risk among all transgender people experiencing these overlapping stigmas.

Youth were eligible if they (1) were aged 16 to 24 years; (2) self-identified as transgender, gender nonconforming, or nonbinary; (3) spoke English (because interviews were conducted in English); (4) identified as Black or African American, Latinx, Asian or Pacific Islander, Indigenous, or multiracial; and (5) lived in Los Angeles. Ultimately, we recruited 108 TGMY between May 25, 2018, and December 7, 2018.

### Recruitment

#### Identifying Appropriate Outreach Methods

On the basis of the lessons learned during recruitment for the HYM Cohort Study, the research staff determined that a wide variety of recruitment techniques would be necessary to reach TGMY of color in the Los Angeles area. Conversations with our community partners, including members of our provider CAB and YCAB as well as service providers at local clinics, informed our methods for connecting with potential participants. In total, 281 potential participants were screened for eligibility, of whom 108 (38.4%) consented to participate in the project and completed a baseline assessment.

One of the difficulties that arose when recruiting TGMY was that it was impossible to know someone’s gender without asking them. At LGBTQIA youth events, any youth may have met the eligibility criteria; therefore, many participants at these events needed to be screened so that we could know more about their identity and experiences. Although there are spaces dedicated to the LGBTQIA community in Los Angeles, the number of spaces intended exclusively for transgender youth is limited. Although LGBTQIA youth events were identified and attended by study staff members, venue-based outreach programs conducted at several of these events and clinics across our 6-month recruitment timeline only accounted for 19.4% (21/108) of the recruited participants.

#### Recruitment Using Social Media

Because of the challenges associated with the in-person recruitment of TGMY, as well as the lessons learned from the recruitment of the HYM Cohort Study, our community partners and research team understood that web-based advertising through social media might be an effective method of recruitment. We used paid advertising with images of gender-diverse youth alongside transgender imagery (such as the transgender pride flag and transgender symbol) to recruit participants on Facebook and Instagram. These sites were identified by our YCAB as places that TGMY frequent and feel most comfortable fully expressing themselves. Participants filled out a screener with their name, demographic information, and contact information. If they met the eligibility criteria for the TRUTH Study, a member of the research field team contacted them to verify their eligibility and invite them to participate in the project. This recruitment strategy accounted for 48.1% (52/108) of the total enrolled participants.

#### Other Recruitment Methods

Participant referrals were recommended by both our CAB and YCAB as an effective strategy for connecting with TGMY, and 24.1% (26/108) of the enrolled participants were recruited using this respondent-driven sampling design. YCAB members and study participants could earn a cash incentive of US $10 for each eligible participant they referred, for up to 5 participants, and US $50 in incentives.

Community partnerships were fostered by the TRUTH Study field team through outreach to LGBTQIA-specific organizations across the Los Angeles area. Specialty clinics in the Los Angeles area served as eligibility screening locations. Several high school and college Genders and Sexualities Alliances were identified, and they expressed interest in disseminating recruitment materials. Recruitment scripts were sent via email to college email listserves as well as community organizations. Although 79 participants were recruited using this community venue–based sampling, only 21 (27%) enrolled into the project. The TRUTH Study also ran advertisements via Craigslist to reach potential participants. Although previous outreach through sites that post sex work–related advertisements has been demonstrated to be successful, the passing of the Stop Enabling Sex Traffickers Act and the Fight Online Sex Trafficking Act and removal of these advertisements from Craigslist and other sites have significantly decreased the activity of these sites. Table 1 presents the recruitment data for each recruitment method.

**Table 1:** Enrollment by recruitment method.^a^

Recruitment method	Participants recruited (N=281), n (%)	Participants enrolled (N=participants recruited by this method), n (%)	Participants enrolled (N=108), n (%)
Physical venue	79 (28.1)	21 (26.6)	21 (19.4)
Social media	142 (50.5)	52 (36.6)	52 (48.1)
Other web-based methods	14 (5)	9 (64.3)	9 (8.3)
Direct referral	46 (16.4)	26 (56.5)	26 (24.1)

^a^Of the 281 potential participants screened for eligibility, 108 (38.4%) consented to participate in the project.

### Tracking and Retention

Longitudinal research conducted with participants experiencing intersecting, stigmatized identities requires specific thoughtfulness and strategies. The TRUTH Study used techniques previously demonstrated to be effective in the HYM Cohort Study for participant tracking and retention [23]. This protocol had been adapted from one used in previous studies to address the complexities of retention with an evolving population in a major metropolitan area [30]. The study protocol included retention strategies such as incentivized monthly check-ins via the participant’s preferred contact method (eg, SMS text message, telephone call, email, Snapchat, and Instagram); additional incentives for contact information updates if participants changed numbers, address, or social media handles; TRUTH Study in-person social events; and STI or HIV test result disclosure and connection to community resources when needed. Our tracking and retention protocol yielded a retention rate of 97.2% (105/108) across 3 waves of data collection.

Community-informed research was essential to building a project that facilitated a safe and inclusive environment for a population with various gender experiences. These steps were important for collecting sensitive information. This involved not only meeting with community members and forming working advisory boards but also hiring research teams with a variety of experiences and identities that represented the population under study. Representation within our field and research staff was necessary to demonstrate our commitment to well-informed practices and to allow our research participants to see themselves reflected within the project team.

A key component of the retention strategy for the TRUTH Study was to pair a field team member with each participant throughout their duration in the project. From the initial eligibility confirmation telephone call to consent visits, study visits, and STI and HIV testing, participants work with the same field team member. This allows the participant to build trust and rapport in the project and ensures that communication about the project is from a single, trusted source. Staff changes may occur; therefore, specific protocols were established to ease the transition. New staff members are introduced by the existing researcher either via the participant’s preferred method of communication (SMS text message, email, etc) or, preferably, during an in-person visit. Consistently updated participant records with contact information, preferred pronouns, insurance status, and day-to-day information help to facilitate these changes as well as eliminate the need for participants to share this information repeatedly with different study staff members.

In addition, the protocol involves obtaining consent to gather multiple forms of contact information. Participants are asked to provide as much of their information as they feel comfortable sharing, including their mobile phone numbers, email addresses, and social media handles, as well as address information, relevant school or work information, and a trusted family or friend contact. Contact information often shifts; therefore, participants are incentivized US $10 to update their contact information with their interviewer, reaching out proactively in the event of a telephone number or address change.

Incentivized monthly check-ins with study staff are built into the study protocol to encourage retention in the study. Between each wave, participants can earn money each month for responding to a check-in inquiry from their assigned field team member. Monthly check-ins are an opportunity for field team members to verify that the contact information is still active and to ask whether participants need referrals to any resources. A rich database of LGBTQIA-specific resources has been created by the field team, and it is available to participants on our study website as well as in an easily distributed PDF file. Participants also use these check-ins as a time to share life updates ranging from difficulties to excitement, such as milestones related to their transition (hormone access or surgery), changes in their access to medical care (through insurance or housing status), or achievements (school graduations and new jobs). If a participant fails to contact their researcher for 2 consecutive check-ins, the provided tracking information and public records (eg, criminal justice records) are used to attempt to reconnect.

Working with multiply marginalized participants highlighted many unique challenges. Our participants needed to be figuratively *met where they were at*. This required the study team to be adaptive and responsive to participants’ needs to reschedule after missing a study visit, extend hours to accommodate participant schedules, and allow for participants to show up late for visits or even walk in for an unscheduled visit. Part of linking 1 research staff member to a participant—for continuity and stability—involved the use of unique mobile phones and numbers for each study staff member. Bidirectional communication between staff members and participants allowed for telephone calls, SMS text messages, emails, and other social media messaging (eg, Snapchat, Facebook Messenger, and Instagram) in a more fluid fashion. As mentioned earlier, participants were incentivized to keep their contact information up to date with their assigned research staff member.

In addition, all staff members on the project were trained in gender affirmation and competency. All research assistants and coordinators were educated to not assume participants’ pronouns or identities, research nurses conducting specimen testing were instructed on using chosen names that may differ from those in hospital records, and policies governing other hospital personnel who interacted with participants were updated to affirm transgender and nonbinary patients more accurately within their systems.

### CAB and YCAB

CABs and YCABs play a critical role in supporting community-partnered and community-informed research. When working with research populations who have been historically denied agency in research narratives about themselves, it is essential to demonstrate accountability and to include community partners in meaningful ways.

Our CAB was formed by inviting members of the HYM Cohort Study CAB and members of service providers and organizations serving gender-diverse populations to an open house to introduce the study. After providing initial feedback, the members were invited back to future meetings to provide guidance and input on the study. CAB members included service providers in the local community, policy makers who focus on transgender issues, STI and HIV test counselors, and medical leads from clinics specializing in transgender individual–related health care. The CAB met bimonthly leading up to the launch of the study and quarterly thereafter.

YCAB members were gender-expansive youth of color recruited from the original HYM Cohort Study as well as youth advocates recommended by existing participants. Study coordinators for the TRUTH Study worked with research assistants on the HYM Cohort Study to identify participants who identified as transgender or nonbinary and invited them to join the YCAB for the TRUTH Study. These YCAB members were also allowed to refer additional members to the YCAB from their social networks. The YCAB met monthly in preparation for the study launch and quarterly during data collection. Before the onset of the COVID-19 pandemic, meetings were held in person, where food as well as stipends worth US $50 were provided to attendees. Meetings moved to the web via videoconferencing software after pandemic lockdown restrictions began. Agendas for the 2 advisory boards often involved study updates, data sharing, data interpretation, and new measure creation. The advisory boards provided information about what data to collect and about the data needs of community organizations. The YCAB provided feedback on proposed new measures and piloted the measures before study launch. Members of the YCAB gave input on which questions could be eliminated to reduce the overall survey length and highlighted and provided suggestions on language that should be updated before being administered to participants.

Our CAB and YCAB were created to provide support and feedback for all components of the study, from eligibility criteria through participant recruitment, including the creation and interpretation of survey measures. It is because of the guidance of the YCAB and CAB that we expanded our eligibility for the project and were able to capture valuable information about TGMY of color, particularly nonbinary and transmasculine youth for whom there is little existing HIV-related research.

### Measures

#### Overview

The TRUTH Study measures were adapted from the protocol previously used to conduct the HYM Cohort Study with YMSM of color in the Los Angeles area [23]. The HYM Cohort Study focused on young sexual minority men of color; therefore, measures had to be adapted to be relevant for a gender-diverse sample of multiple sexual identities. Our CAB and YCAB were instrumental in providing guidance on how to restate phrases in the original measures; for example, “attraction to men” in previous measures was changed to “LGBTQ identity.” In addition, meetings with the YCAB and CAB identified additional constructs relevant to gender-diverse youth that were not assessed in the HYM Cohort Study. For these constructs, additional measures were either adapted (if not written for gender-diverse populations), added (if already constructed for gender-diverse populations), or created by the study team in conjunction with the YCAB and CAB when no existing measures were found.

TRUTH Study participants completed 3 study visits, with consecutive visits spaced 6 months apart. Study visits consisted of self-report survey measures, urine collection for substance screening, biorepository specimen collection, and STI and HIV testing. Consistent with the HYM Cohort Study, survey measures were administered by research assistants, with the more sensitive topics being self-administered using a web-based survey (eg, STI and HIV testing results history, sexual behaviors, substance use, suicidality, and gender-based discrimination); the goal was to provide additional confidentiality and to encourage honesty in responses [31,32]. The interview and self-administered survey required approximately 90 minutes to complete. Participants received US $105 to compensate them for their time and effort completing all study procedures. A description of the study measures follows.

#### Demographic Characteristics

Survey measures to obtain demographic information were modeled from the HYM Cohort Study protocol. Information collected included age, language spoken inside and outside the home, race and ethnicity, religion, residential stability, educational history and current employment status, access to food and food security, incarceration, and foster care experience [23]. Updates to the methods used to obtain demographic data were influenced by our CAB and YCAB members to ensure care when gathering demographic data from participants with intersecting identities; for instance, TRUTH Study participants reported multiple gender identities, racial and ethnic group memberships, and sexual identities and orientations across all waves of data collection rather than being forced to choose only one identity facet.

#### Primary Outcome Measures

##### Alcohol, Tobacco, Marijuana, and Illicit Drug Use

Substance use within the TRUTH Study was measured using the same scales and assessments as the HYM Cohort Study protocol [23]. TRUTH Study participants completed self-administered scales to assess lifetime, past 6-month, and past 30-day substance use as well as biometric urine screening for substances at each study visit. These scales are from the Monitoring the Future and 2014 National Survey on Drug Use and Health studies and ask about use of alcohol, nicotine, marijuana, lysergic acid diethylamide, phencyclidine, mushrooms, cocaine, crack, methamphetamines, ecstasy, stimulants, heroin, fentanyl, poppers, and prescription drugs used without a physician’s order [33]. The scales address frequency of use as well as location, circumstances, and substance use associated with sexual behaviors. Additional questions were added to these scales pertaining to shared needle use regarding hormones and bodily injections, such as silicone.

The point-of-care biometric urinalysis test administered at survey visits used the Integrated E-Z Split Key Cup II-10 Panel (Alere Toxicology) that measures metabolites of amphetamines, methamphetamines, benzodiazepines, cocaine, ecstasy, phencyclidine, methadone, fentanyl, opiates, and marijuana. This test can detect marijuana use for up to 30 days and other drugs from 1 to 4 days after use [34,35]. Participants could opt to receive a copy of their substance use results during their study visit.

##### Problem Alcohol and Marijuana Use

Alcohol and marijuana misuse was assessed using standardized measures, including the Alcohol Use Disorders Identification Test [36,37], which assesses frequency of participants’ alcohol use, and items from the Diagnostic and Statistical Manual of Mental Disorders, Fourth Edition [38], and Diagnostic and Statistical Manual of Mental Disorders, Fifth Edition [39], which assess marijuana use and its associated life impacts.

##### Sexual Activity, Partners, and HIV Risk and Protective Behaviors

Survey measures looking at sexual activity, number and genders of sexual partners, and use of protective factors such as PrEP and condoms were adapted from the HYM Cohort Study protocol scales. The TRUTH Study team worked to generate affirming methods of measuring sexual activity for transgender and nonbinary participants because the HYM Cohort Study scales were adapted from the EXPLORE study specifically for YMSM [35,40]. As little is known about the sexual risk factors for nonbinary young people, care and consideration should be taken in adapting sexual activity scales for transgender and nonbinary populations so that adequate data are collected while respecting participants. On the basis of feedback from our CAB and YCAB members, options were added to increase participant comfort in answering sexual activity–related questions. Participants were prompted to enter the words they used to describe *penis* and *vagina*, and these words were populated into the survey module. This functioned to affirm participants’ own language for their bodies and the bodies of their sexual partners, as well as to avoid distress related to dysphoria around gendered words for body parts. Additional types of partners and types of sexual interactions also needed to be added to the questionnaires. Participants preferred to be able to choose their partner’s specific gender (such as *genderqueer* or *trans femme*) as opposed to choosing from *male, female, or intersex*. The specific questions used to assess sexual activity and partners are outlined in the HYM Cohort Study protocol [23].

HIV and PrEP knowledge was assessed in the self-administered STI and HIV testing section of the survey. HIV knowledge was assessed using a 2-part question from Hou et al [41] as well as a 15-point scale about status knowledge and treatment-based beliefs created by Kalichman et al [42]. PrEP knowledge and willingness were measured using a 10-item scale from Grov et al [43]. PrEP and HIV-related treatments were discussed with participants during the HIV testing process as per Los Angeles county HIV test counselor guidelines.

##### STI and HIV History and Test Results

Participants self-reported their lifetime and recent history of HIV and STI testing and HIV status. This section of the survey asked about testing behaviors, HIV and STI testing results, access to treatment, as well as any hesitance around HIV and STI testing. Condom use across partners as well as condom self-efficacy were also measured [44]. Participants were also asked whether they had exchanged sex for things such as money, hormones, transportation, or a place to stay ever in their lifetime and within the last 6 months.

Research staff members administered rapid HIV testing and a complete STI testing panel to participants at each study visit. HIV and STI testing protocols followed the methods used for the HYM Cohort Study [23], with the addition of optional site-specific vaginal swab specimen collection and gender-affirming testing administration instructions. HIV status was measured using a point-of-care whole blood finger-stick device. Participants self-collect vaginal or frontal, rectal, and pharyngeal specimens for *Neisseria gonorrheae* and *Chlamydia trachomatis* nucleic acid amplification. Syphilis testing was conducted using whole blood samples collected via venipuncture using rapid plasma regain and treponemal antibody testing. All research staff and nursing staff members associated with the project underwent an LGBTQIA health care training to be able to provide compassionate care.

Participants with positive test results were connected to members of the research staff certified in HIV and STI test counseling and then referred to an appropriate community clinical partner for treatment. Research staff members were available to participants via SMS text message, telephone, and email to assist with accessing care.

#### Measures of Overall Health, Health Care Access, and Mental Health

Overall health, well-being, and access to health care were measured using scales adapted from those used with the HYM Cohort Study [23]. Questions about insurance coverage, access to a primary health clinic, number of visits in the past 12 months, and reasons for health care visits were measured using items from the National Longitudinal Study of Adolescent to Adult Health and the National Survey of Children’s Health [45]. Participants were asked about their perception of their own health, any chronic health conditions (including mental health diagnoses), and the level of impact these health conditions had on their everyday functional abilities. These scales were adapted to include questions about hesitance to seek care because of perceived gender-based discrimination, comfort discussing gender and sexual questions with a provider, disclosing one’s gender to one’s provider, and how often clinicians used correct pronouns. The importance of being seen in an LGBTQIA-specific clinic was also measured. Hormone and other bodily injection use (such as silicone) for gender presentation was assessed at each visit. This questionnaire asked about current hormone or injection use, frequency of use, source of hormones (prescription, a friend, or the internet), method of use, and method of administration (self-injection, nurse injection, etc). Access to mental health care providers to write letters for gender-affirming procedures such as hormones or surgery was assessed.

#### Possible Mediating and Moderating Constructs

##### Overview

Although we hoped to capture the mental well-being of the TRUTH Study cohort using clinical assessments, we also included measures that were hypothesized to be protective factors against negative mental health outcomes. The protective factors included optimism, resilience, and mindfulness. Assessments of these factors were adapted from the HYM Cohort Study protocol with attention to possible life experiences specific to TGMY [23].

##### Mental Health, Emotion Regulation and Coping, Optimism, Resilience, and Mindfulness

Depression, anxiety, and somatization were measured using the 18-item Brief Symptom Inventory, which asks participants to rate, using a 5-point scale, how extensively a symptom or trait has bothered them in the last week [46]. Lifetime, past 6-month, and recent self-injury and suicidality were measured in a multistep, escalating scale (ie, questions about suicidal thought, ideation, and attempts were asked). Participants who responded yes to these questions were connected to transgender individual–specific mental health resources by their assigned research assistant.

##### Childhood Abuse and Trauma, Stressful Life Events, and IPV

Childhood abuse and trauma experiences were measured by the Bernstein Childhood Trauma Questionnaire [47]. IPV was measured using a 14-point scale adapted from Straus et al [48]. This scale measures both victimization and perpetration of IPV and specifically cites modern interactions that young people have, such as controlling social media or mobile phone use [49].

A 43-item stressful life events scale was adapted from the HYM Cohort Study [50] and updated to include items related to transgender individual–specific experiences. This scale asks participants whether they had experienced a stressful life event, and if yes, to rate the amount of stress that this event caused on a 10-point scale. Some of the adapted questions involved family arguments over gender, coming out to family members or friends, or losing a friend because of transitioning.

##### Social Support

Perception of general social support among family, friends, and a *special person* (such as partner or close friend) was measured using a 12-item scale [51]. Social support specifically relating to participants’ transgender identity, such as support from family members, friends, and social belongingness within LGBTQIA communities, was measured through 4 questions adapted from Bockting et al [52].

##### Racism, Transphobia, and Discrimination

Perception of stigma against transgender and gender nonbinary people was measured using a 6-item scale asking participants to rank how they perceive other people’s reactions to transgender people. This scale has been adapted for transgender and gender minority populations [53].

Participants complete a self-administered 54-item scale regarding the interactions they have had of lifetime and recent racism [54], transphobia, discrimination, and harassment [55]. During the second wave of data collection, we used a retrospective bullying questionnaire from Hamburger et al [56] to ask participants about their experiences across childhood, teenage years, and into young adulthood.

##### Positive Transgender Belongingness and Positive Transgender Identity

Positive sense of self and perceived belongingness within the transgender community were also assessed. Positive transgender identity was measured using a 24-item scale adapted from a scale previously used to measure positive LGBTQIA identity and community belongingness. Questions such as “I embrace my identity” and “I feel a connection to the community” were asked on a 5-point scale. The measure can be scaled to measure different factors associated with positive self-identity, such as authenticity, relationships, commitment to social justice, and self-acceptance and awareness [57].

##### Homegrown Scales

In collaboration with our YCAB and CAB members, we convened regular meetings to determine what research questions would be most beneficial and influential to the community. Feedback from our meetings led to the creation of several new scales. To capture the differing experiences of comfort being *out* to others about their gender identity, several questions were used to assess the age at which the participants first identified as transgender or felt that they had a gender identity that did not align with the sex assigned to them at birth, when they first told someone about their gender, and how often they share about their gender with others. Participants were asked about their pronouns and possible shifts in pronoun use; appropriate pronoun use by friends, family, coworkers, and community members; and how often they had been asked about their pronouns. They were also asked about experiences of gender affirmation and how they felt about achieving affirmation through clothing, social cues, medical procedures, and spirituality.

#### Qualitative Questions

Our YCAB encouraged us to use open-ended questions at the end of the second and third waves of data collection. Because of the unique experiences of TGMY of color and their intersectional experiences, the YCAB members believed that open-ended questions would allow for more expression and nuance from participants. In collaboration with the YCAB, the research team developed the following open-ended questions:

1. How has the TGMY community empowered you?
2. What makes you euphoric in your gender?

#### Biological Specimens and Biorepository

TRUTH Study participants consented to providing biological samples (10 mL ethylenediamine tetra-acetic acid anticoagulated whole blood sample and 1 rectal swab) for our biorepository during one of their study visits. Samples were stored in a freezer maintained at a temperature of –80 °F (–62 °C) for future use.

## Results

The TRUTH Study cohort was recruited between May 25, 2018, and December 7, 2018. Three waves of data collection occurred between May 25, 2018, and April 9, 2020. Baseline data were presented at the Center for HIV Identification, Prevention, and Treatment Services annual conference in January 2020. Baseline and longitudinal data are being analyzed as of August 2022. In July 2020, we received additional funding from the National Institutes of Health (5U01DA036926) to conduct additional waves of data collection across 5 more years with our existing cohort and to recruit 250 additional TGMY of color to participate. The study team leadership met in September 2022 to review the performance of the measures—especially those measures that were adapted or newly created—and to make recommendations for future measures to include in future waves. The team decided to retain new or adapted measures for at least one additional wave to assess performance over time.

## Discussion

### Overview

This paper describes the addition of a gender-diverse sample complementary to the sample in the HYM Cohort Study [23]. We discuss the process of forming 2 community boards to advise recruitment, enrollment, marketing materials, study measures, and study design. Further, we discuss our methods for adapting measures developed for sexual minority men and other populations to be sensitive to, and relevant for, TGMY of color. The longitudinal nature of the TRUTH Study allows us to augment questions at each wave to address historical and structural changes that may affect the lives of TGMY of color as well as gender-diverse youth of color; for example, multiple global pandemics and a shifting policy landscape.

Transgender and gender minority youth, particularly those of color, exist at the intersection of multiple complex identities and experiences. From public health reform and social justice advocacy to youth empowerment and positive self-identity, it is imperative to consider the strategies needed to address disparities in health care and other social determinants that place TGMY of color at risk for negative health outcomes. Research conducted with TGMY of color needs to acknowledge the historical hierarchical structures that exist to gatekeep individuals from appropriate care and empowerment and work to heal this through community-informed research practices created by, and for, transgender people.

Although our sample experiences a host of systemic barriers, through the guidance of our advisory boards and by engaging in appropriate gender affirmation trainings we were able to create a space that allowed for a retention rate of 97.2% (105/108) across the first 3 waves of data collection. Our advisory boards provided instrumental feedback on where to recruit participants, what data to collect, and how to assess constructs in the survey. Our approach of connecting participants to a single staff member for communication and study visits provided stability across the life of the study. Staff members were provided with mobile phones, which allowed them to have a single source by which participants could contact them to provide updates and stay connected between study visits. We believe that these strategies combined to help us achieve a high level of retention.

### Inclusive and Nonjudgmental Measures

Researchers looking to engage participants with multiply marginalized identities should strive to ensure that their research procedures do not accidentally further victimize the participants. Making sure that the measures are inclusive and nonjudgmental can reduce stress during the survey process. Any person who may interact with participants during the study process (including those outside the study team) should be trained to understand how the language they use and the tasks they perform may affect participants. Only then can we begin to build trust between research and multiply marginalized communities.

## Abbreviations

CAB: community advisory board
HYM: Healthy Young Men’s
IPV: intimate partner violence
LGBTQIA: lesbian, gay, bisexual, transgender, queer, intersex, and asexual
PrEP: pre-exposure prophylaxis
STI: sexually transmitted infection
TGMY: transgender minority youth
TRUTH: Trans Youth of Color
YCAB: youth community advisory board
YMSM: young men who have sex with men

## Data Availability

Data from this study are available upon email request to the senior author (MDK). To enhance participant confidentiality, any data users must go through a review process and sign a data use agreement with the institution responsible for data safety before receiving data from the Trans Youth of Color Study.
